# Modeling brain circuitry over a wide range of scales

**DOI:** 10.3389/fnana.2015.00042

**Published:** 2015-04-07

**Authors:** Pascal Fua, Graham W. Knott

**Affiliations:** ^1^Computer Vision Lab, I&C School, École Polytechnique Fédérale de LausanneLausanne, Switzerland; ^2^Bioelectron Microscopy Core Facility, École Polytechnique Fédérale de LausanneLausanne, Switzerland

**Keywords:** delineation, segmentation, connectomics, mitochondria, synapses, dendritic arbors

## Abstract

If we are ever to unravel the mysteries of brain function at its most fundamental level, we will need a precise understanding of how its component neurons connect to each other. Electron Microscopes (EM) can now provide the nanometer resolution that is needed to image synapses, and therefore connections, while Light Microscopes (LM) see at the micrometer resolution required to model the 3D structure of the dendritic network. Since both the topology and the connection strength are integral parts of the brain's wiring diagram, being able to combine these two modalities is critically important. In fact, these microscopes now routinely produce high-resolution imagery in such large quantities that the bottleneck becomes automated processing and interpretation, which is needed for such data to be exploited to its full potential. In this paper, we briefly review the Computer Vision techniques we have developed at EPFL to address this need. They include delineating dendritic arbors from LM imagery, segmenting organelles from EM, and combining the two into a consistent representation.

## 1. Introduction

**Figure 1:**
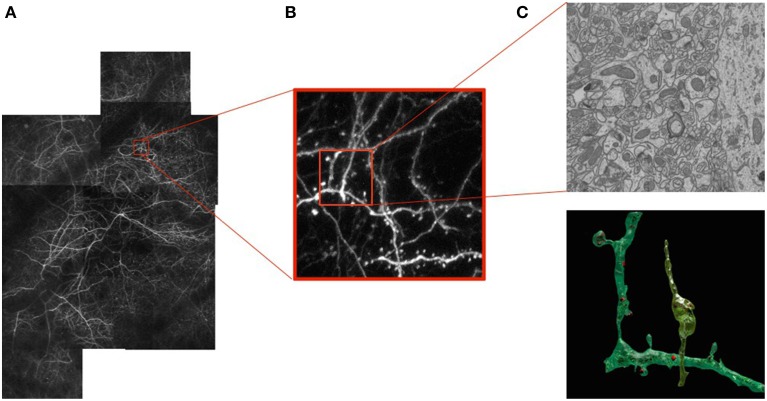
**Correlative Microscopy. (A)** Fluorescent neurons *in vivo* in the adult mouse brain imaged through a cranial window. **(B)** Image stack at the 1 μm resolution acquired using a 2-photon microscope. **(C)** Image slice of a sub-volume at the 5 nm resolution above a reconstruction of a neuron, dendrite, and associated organelles.

As our ability to image neurons with light and electron microscopes improves, so does our understanding of their form and function. Today we can image large volumes of both live and fixed brain tissue across a wide range of resolutions. At the micrometer scale, light microscopy (LM) of fluorescently labeled structures reveals dendrites and axons of a subset of neurons that can potentially be reconstructed revealing their complex 3D network, as shown in Figure 1B. However, their internal structures and all their surrounding elements remain invisible when using this technique. To see them, one must turn to electron microscopes (EM). These provide images at the nanometer scale making it possible to visualize all the structural elements and especially those that are important for understanding the basic connectivity and activity of different cells. These include synapses, dendritic spines, vesicles, and mitochondria, as depicted by Figure 1C.

These recent technologies will therefore provide crucial information about the structural, functional, and plasticity principles that govern neural circuits. And since most neurological and psychiatric disorders involve deviations from these principles, such an understanding is key to treating them. Furthermore, neural circuits exhibit a computational power that no known technology can match. A more thorough understanding of their complexities could therefore spur development of new paradigms and bio-inspired devices that would far outperform existing ones.

However, a major bottleneck stands in the way of this promise: These new microscopes can produce terabytes upon terabytes of image data that is so rich and so complex that humans cannot analyze them effectively in their entirety. In this paper, we will briefly present the algorithms we have developed at EPFL to automatically recover the dendritic and axonal trees, segment intra-neuronal structures from EM images, and register the resulting models. For further details, we refer the interested reader to the original publications.

## 2. Delineation

The automated delineation of curvilinear structures has been investigated since the inception of the field of Computer Vision in the 1960s and 1970s. Nevertheless, despite decades of sustained effort, full automation remains elusive when the image data is as noisy and the structures exhibit as complex a morphology as they do in microscopy data. As a result, practical systems still require extensive manual intervention that is both time-consuming and tedious. For example, in the DIADEM challenge to map nerve cells, the results of all the finalists still required substantial time and effort to proofread and correct Ascoli et al. (2010); Peng et al. (2011).

Part of the problem comes from the fact that many existing techniques rely mostly on weak local image evidence, and employ greedy heuristics that can easily get trapped in local minima. As a result, they lack robustness to imaging noise and artifacts. Another common issue is that curvilinear networks are usually treated as tree-like structures without any loops. In practice, however, many interesting networks are not trees since they contain cycles. Furthermore, even among those that really are trees, such as neurites, the imaging resolution is often so low that the branches appear to cross, thus introducing several spurious cycles that can only be recognized once the whole structure has been recovered. In fact, this is reported as one of the major sources of error in Bas and Erdogmus (2011); Chothani et al. (2011); Turetken et al. (2011); Wang et al. (2011); Zhao et al. (2011); Choromanska et al. (2012) and a number of heuristics have been proposed to avoid spurious connections in Chothani et al. (2011); Turetken et al. (2011); Zhao et al. (2011).

### 2.1. Approach

**Figure 2:**
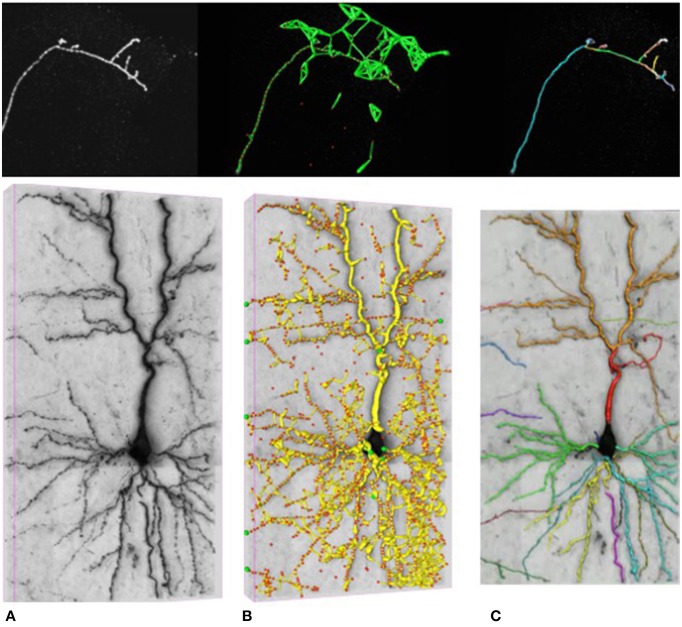
**Delineation in confocal (top) and brightfield (bottom) imagery. (A)** The original 3D stacks. **(B)** The nodes appear as red circles with the tubular paths connecting them overlaid in green and yellow. **(C)** The final 3D delineations.

In our work, we attempt to overcome these limitations by formulating the reconstruction problem as one of solving an Integer Program (IP) on a graph of potential tubular paths. As shown in Figure 2, the resulting algorithm goes through the following steps:

- We first compute a *tubularity* value at each image location and radius value. It quantifies the likelihood that there exists a tubular structure of this radius at that location. Given an 3D stack, this creates an 4D scale-space tubularity volume.
- We select regularly spaced high-tubularity points as seed points and connect pairs of them that are within a given distance from each other. This results in a directed tubular graph, such as those shown in Figure 2B, which serves as an overcomplete representation for the underlying curvilinear networks.
- Having trained a path classifier using such graphs and ground-truth delineations, we assign probabilistic weights to pairs of consecutive edges of a given graph at detection time.
- We use these weights and solve an integer program to compute the maximum-likelihood directed subgraph of this graph to produce a final result such as the one of Figure 2C.

These four steps come in roughly the same sequence as those used in most algorithms that build trees from seed points, as in Fischler et al. (1981); Turetken et al. (2011); Wang et al. (2011); Zhao et al. (2011), but with three key differences. First, whereas heuristic optimization algorithms such as MST followed by pruning or the k-MST algorithm of Turetken et al. (2011) offer no guarantee of optimality, our approach guarantees that the solution is within a small tolerance of the global optimum. Second, our approach to scoring individual paths using a classifier instead of integrating pixel values as usually done gives us more robustness to image noise and provides peaky probability distributions, which helps ensure that the global optimum is close to the ground truth. Finally, instead of constraining the subgraph to be a tree as many state-of-the-art approaches, we allow it to contain cycles and instead penalize spurious junctions and early branch terminations as described in more details in Turetken et al. (2012, 2013a).

### 2.2. Results

Here, we demonstrate the effectiveness of our approach on the two datasets depicted in Figure 2:

- *Confocal-Axons*, 8 image stacks of Olfactory Projection Fibers (OPF) of the Drosophila fly acquired using a 3D confocal microscope and taken from the DIADEM competition.
- *Brightfield*: 6 image stacks were acquired by brightfield microscopy from biocytin-stained rat brains.

In both datasets, the neurites form tree structures without cycles. However, in the latter, disjoint branches appear to cross, introducing false loops, due to the low z-resolution. In both cases, we used half the stacks for training and half for testing. We used a semi-automated delineation tool Turetken et al. (2013b) to extract ground truth tracings from the training stacks and train our path-classifiers.

In Table 1, we compare our approach (OURS) to several state-of-the-art algorithms on the confocal-axons. They are the pruning-based approach (APP2) of Xiao et al. (2013), the active contour algorithm (OSnake) of Wang et al. (2011), the NeuronStudio (NS) software of Wearne et al. (2005), the focus-based depth estimation method (Focus) of Narayanaswamy et al. (2011), and finally the k-MST technique of Turetken et al. (2011), the last two of which were finalists in the DIADEM competition. For all these algorithms, we used the implementations provided by their respective authors with default parameters. We report DIADEM scores as described in Ascoli et al. (2010), which were designed to compare topological accuracy of a reconstructed tree against a ground truth tree.

**Table 1:** **DIADEM Ascoli et al. (2010) scores on four test stacks from the Confocal-Axons dataset**.

	**OURS**	**k-MST Turetken et al. (2011)**	**NS Wearne et al. (2005)**	**OSnake Wang et al. (2011)**	**APP2 Xiao et al. (2013)**
OPF4	**0.91**	0.87	0.58	0.00	0.67
OPF6	**0.91**	0.90	0.65	0.80	0.82
OPF7	**0.94**	0.91	0.42	0.68	0.76
OPF8	**0.90**	0.74	0.58	0.69	0.63

*Each row corresponds to an image stack denoted by OP*i*. Higher scores are better*.

We also evaluated the APP2 Xiao et al. (2013), OSnake Wang et al. (2011), and Focus Narayanaswamy et al. (2011) algorithms on the Brightfield dataset. Since they do not allow the user to provide multiple root vertices, the DIADEM score of their output cannot be computed. To compare their algorithms to ours, we therefore used the NetMets measure of Mayerich et al. (2012) instead because it does not rely heavily on roots. As the DIADEM metric, this measure takes as input the reconstruction and the corresponding ground truth tracings. However, it is more local because it does not account for network topology.

Table 2 shows the NetMets scores on the test images of the *Brightfield* dataset. Note that the Focus algorithm of Narayanaswamy et al. (2011) is specifically designed for brightfield image stacks distorted by a point spread function. Our approach nevertheless brings about a systematic improvement except in one case (BRF3—connectivity FPR). However, the algorithm does that best in this category does significantly worse in the other three.

**Table 2:** **NetMets Mayerich et al. (2012) scores on the Brightfield dataset**.

**OURS**	**BRF1**				**BRF2**				**BRF3**			
	**0.05**	**0.29**	**0.71**	**0.65**	**0.11**	**0.29**	**0.81**	**0.78**	**0.07**	**0.28**	0.77	**0.70**
k-MST Turetken et al. (2011)	0.10	0.44	0.79	0.88	**0.11**	0.53	0.84	0.91	0.13	0.35	0.81	0.92
Focus Narayanaswamy et al. (2011)	0.39	0.54	0.75	1.00	0.49	0.53	0.90	1.00	0.38	0.46	**0.74**	1.00
OSnake Wang et al. (2011)	0.66	0.63	0.98	0.99	0.66	0.59	0.99	1.00	0.69	0.38	0.95	0.99
APP2 Xiao et al. (2013)	0.68	0.64	1.00	1.00	0.63	0.54	1.00	1.00	0.65	0.49	1.00	1.00

*The NetMets software outputs four values for each trial, which are geometric False Positive Rate (FPR), geometric False Negative Rate (FNR), connectivity FPR, and connectivity FNR, respectively from left to right. Lower scores are better. The best scores are shown in bold face*.

## 3. Segmentation

To observe the connectivity between neurons electron microscopy is required. In our work, we have used Focus Ion Beam Scanning Electron Microscopy (FIBSEM) at a 5 nm nearly isotropic sampling. The resulting image stacks reveal the fine neuronal structures, including the synaptic contacts. However, segmenting EM data poses unique challenges in part because the volumes are heavily cluttered with structures that exhibit similar textures and are therefore difficult to distinguish based solely on local image statistics. In this section, we outline our approach to segmenting both synapses and mitochondria. They are described in more details in Becker et al. (2013); Lucchi et al. (2014).

### 3.1. Synapses

#### 3.1.1. Approach

Synapses are difficult to distinguish from other structures based solely on local texture, as shown in **Figure 4**. Human experts confirm their presence by looking for nearby for post-synaptic densities and vesicles. This protocol cannot be emulated simply by measuring filter responses at the target voxel as in Kreshuk et al. (2011), pooling features into a global histogram as in Narasimha et al. (2009); Lucchi et al. (2012) or relying on hand-determined locations for feature extraction as in Venkataraju et al. (2009); Jurrus et al. (2010).

**Figure 3:**
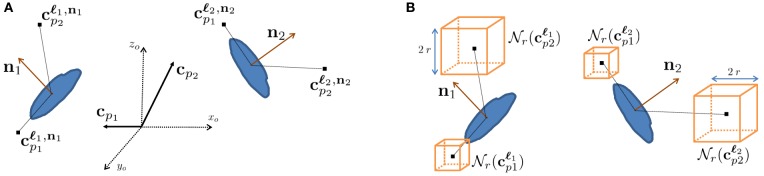
**Context features. (A)** Relative context cue locations **c**_*p*_ in the global coordinate system *x_o_, y_o_, z_o_* are rotated according to the orientation estimate of the voxel of interest **n**_*i*_ to yield locations **c**^**ℓ**_*i*_^_*p*_ that are consistent. **(B)** At each of these locations, image channels are summed over cubes of radius *r* around their center. Our approach employs AdaBoost to select the most discriminative features for synapse segmentation.

To emulate this human ability, we designed features we call *context features*, which can be extracted in any cube contained within a large volume centered on the voxel to be classified at 3D location **ℓ**_*i*_ with local orientation **n**_*i*_, as depicted in Figure 3. They are computed in several image channels using a number of Gaussian kernels. This yields more than 100,000 potential features and we rely on AdaBoost to select the most discriminative ones.

#### 3.1.2. Results

**Figure 4:**
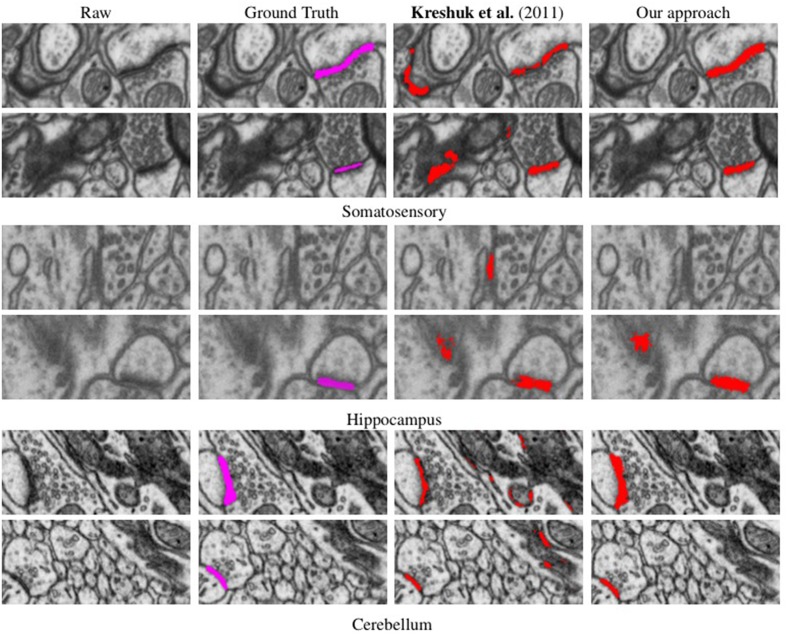
**Synapse segmentations overlaid on individual slices from three different datasets after thresholding**. Note that our approach yields more accurate results than the method of Kreshuk et al. (2011) with almost no false positives.

We evaluated our method on three different EM stacks acquired from different regions of the adult rat brain, the Somatosensory Cortex, the Hippocampus, and the Cerebellum. Example slices from each dataset are shown in Figure 4 along with our results.

**Figure 5:**
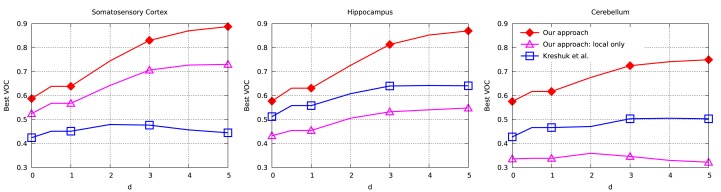
**Jaccard index (VOC score) as a function of exclusion zone size *d* for the different datasets**. Our approach outperforms Kreshuk et al. (2011) for all values of *d*.

To evaluate the performance of our approach and compare it to that of Kreshuk et al. (2011), we performed a voxel-wise evaluation against manually acquired ground-truth data. To discount the influence of boundary voxels whose classification may be ambiguous, we defined a testing exclusion zone around the labeled border of the synapse within a distance of *d*. The voxels within that exclusion zone are ignored and, in Figure 5, we plot the Jaccard index between the ground-truth labeling and the one the two algorithms produce as a function of *d*. To highlight the importance of using context, we plot a third curve that correspond to our approach using only boxes centered on the voxel to be classified, which is much worse than the other two.

### 3.2. Mitochondria

Mitochondria participate in a wide range of cellular functions and their morphology and localization play a key role in cellular physiology Campello and Scorrano (2010). Furthermore, localization and morphology of mitochondria have been tightly linked to neural functionality. For example, pre- and post-synaptic presence of mitochondria is known to have an important role in synaptic function, as shown in Lee et al. (2007), and mounting evidence also indicates a close link between mitochondrial function and many neuro-degenerative diseases Knott et al. (2008); Poole et al. (2008).

New approaches to detecting mitochondria in EM images have therefore begun to appear. For example, in Vitaladevuni et al. (2008) a Gentle-Boost classifier was trained to detect them based on textural features. In Narasimha et al. (2009), texton-based mitochondria classification in melanoma cells was performed using a variety of classifiers including k-NN, SVM, and Adaboost. While these techniques achieve reasonable results, they incorporate only textural cues while ignoring shape information. More recently, more sophisticated features have been successfully used in Kumar et al. (2010); Sommer et al. (2010); Lucchi et al. (2012) in conjunction with either a Random Forest classifier as in Kreshuk et al. (2011). The algorithm of Marquez-Neila et al. (2014) could be used to impose higher-order shape constraints but would be very difficult to extend to 3D volume segmentation because its computational requirements are prohibitive. Our approach overcomes this limitation and extends these earlier techniques by explicitly modeling membranes and exploiting the power of our context features in a Structured SVM framework Lucchi et al. (2014).

#### 3.2.1. Approach

**Figure 6:**
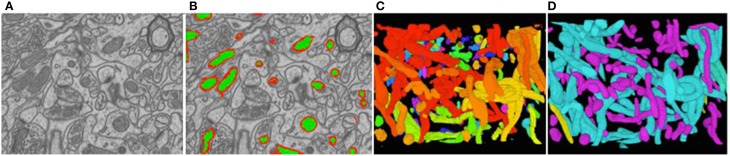
**Reconstructed mitochondria. (A)** Slice from a 3D image stack. **(B)** The inside of the mitochondria are overlaid in green and the membranes in red. **(C)** Raw results. **(D)** Edited results. The dendritic mitochondria are shown in cyan and axonal ones in purple.

To reduce the computational complexity, our first step of our approach is to over-segment the image stack into *supervoxels*, that is, small voxel clusters with similar intensities. We use the algorithm of Achanta et al. (2012) to compute them. It lets us choose their approximate diameter, which we take to be on the order of the known thickness of the outer mitochondrial membranes. This means that membranes are typically one supervoxel thick. All subsequent computations are performed on supervoxels instead of individual voxels, which speeds them up by several orders of magnitude. Our task is now to classify these supervoxels as being inside the mitochondria, part of the membrane, or outside, as shown in Figure 6B.

To this end, we introduce a three-class Conditional Random Field (CRF) Lafferty et al. (2001). It is defined over a graph 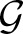 = (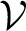, 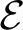) whose nodes *i* ∈ 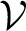 correspond to supervoxels and whose edges (*i, j*) ∈ 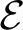 connect nodes *i* and *j* if they are adjacent in the 3D volume. Each node is associated to a feature vector *x_i_* computed from the image data and a label *y_i_* denoting one of the three classes to which a supervoxel can belong. Let *Y* be the vector of all *y_i_*, which we will refer to as a *labeling*. The most likely labeling of a volume is then found by minimizing an objective function of the form

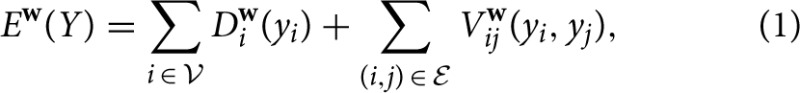



where *D_i_* is referred to as the unary data term and *V_ij_* as the pairwise term. The superscript denotes the dependency of these two terms to a parameter vector **w**.

The unary data term *D_i_* is taken to be a kernelized function of the context features of Section 3.1.1. The pairwise term is a linear combination of a spatial regularization term and a containment term. The spatial term is learned from data and reflects the transition cost between nodes *i* and *j* from label *y_i_* to label *y_j_*. The containment term constrains the membrane class to completely enclose the inside class and to be at least one supervoxel thick, as originally proposed in Delong and Boykov (2009). This containment term is hand-defined and does not depend on any parameters. The set of parameters **w** to be learned are therefore the weights given to individual features in the unary term and the spatial regularization term. These parameters are learned within the Structured SVM framework discussed above, which requires solving an inference problem on the supervoxel graph 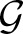.

#### 3.2.2. Results

Figure 6C depicts the 3D reconstructions we obtained from a 3.21 × *m* × 3.21 μ*m* × 1.08 μ*m* volume. In Figure 6D, we show the same results after having been proof-read and hand-corrected by a trained neuroscientist. The whole process, including generating the training data, took a little under 2 h. For comparison purposes, the neuroscientist re-generated these results entirely manually and that took him about 6 h for a similar level of precision in terms of the mitochondria volumes and surface areas, which are the relevant biological quantities. In other words, automation reduced the required amount of manual intervention by a factor 3. Going further will require deploying tools based on deformable models such as those of Neuenschwander et al. (1994, 1997); Jorstad and Fua (2014) to automatically refine mitochondria boundaries and break apart incorrectly merged ones.

To further quantify the performance of our approach, we compared it against other recent automatic methods on image stacks from the Hippocampus and Striatum, which are similar to those we used to detect synapses. In Table 3, we report the Jaccard index for the foreground and membrane class jointly, which is representative for this task since whole mitochondria are the object of interest being segmented. The first one is a very recent mitochondria segmentation method Seyedhosseini et al. (2013) that does *not* rely on structured learning. Instead, it trains a cascade of classifiers at different scales and has been shown to outperform earlier algorithms based on Neural Networks, SVMs, and Random Forests on EM imagery. The others correspond to different approaches to performing structured learning. As can be seen, we consistently outperform the competing methods.

**Table 3:** **Comparing segmentation performance as measured by the Jaccard index of the foreground class for the Striatum and Hippocampus datasets against that of a number of baselines**.

	**Seyedhosseini et al. (2013) (%)**	**Tsochantaridis et al. (2004) (%)**	**Wick et al. (2011) (%)**	**Lacoste-Julien et al. (2013) (%)**	**Ratliff et al. (2007) (%)**	**OURS (%)**
Hippocampus	83.8	92.7	83.3	92.7	89.2	**94.8**
Striatum	83.5	90.6	89.6	90.5	88.1	**92.1**

*The best scores are shown in bold face*.

## 4. Registration

Registering LM and EM stacks such as those of Figures 1B,C is required to identify the same region in both images and to combine the specific information each modality provides, as discussed earlier. However, this is challenging because the scale-discrepancy between the two modalities—1000 nm for EM vs. 5 nm for LM—produces drastic appearance changes. It makes it impractical to use standard registration techniques that rely on maximizing image similarity, such as those described in Pluim et al. (2003).

Instead, we have proposed in Serradell et al. (2015) a new approach for matching graph structures embedded in 3D volumes, which can deal with the scale-change while being robust to topological differences between the two graphs and even changes in the distances between vertices, unlike earlier graph-matching techniques such as those of Deng et al. (2010); Smeets et al. (2010). It requires no initial position estimate, can handle non-linear deformations, and does not rely on local appearance or global distance matrices. Instead, given graphs extracted from the two images or image-stacks to be registered, we treat graph nodes as the features to be matched. We model the geometric mapping from one data set to the other as a Gaussian Process whose predictions are progressively refined as more correspondences are added. These predictions are in turn used to explore the set of all possible correspondences starting with the most likely ones, which allows convergence at an acceptable computational cost even though no appearance information is available.

### 4.1. Approach

Given graphs 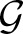
^*A*^ = (**X**^*A*^, **E**^*A*^) and 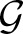
^*B*^ = (**X**^*B*^, **E**^*B*^) extracted from image-stacks *A* and *B*, let the **E**s denote edges and the **X**s nodes. The edges, in turn, are represented by dense sets of points forming 3D paths connecting the nodes. Our goal is to use these two graphs to find a geometrical mapping *m* from *A* to *B* such that *m*(**x**^*A*^_*i*_) is as close as possible to **x**^*B*^_*j*_ in the least-squares sense assuming that **x**^*A*^_*i*_ and **x**^*B*^_*j*_ are corresponding voxels.

If correspondences between points belonging to the two graphs were given, we could directly use the Gaussian Process Regression (GPR) as in Rasmussen and Williams (2006) to estimate a non-linear mapping that would yield a prediction of *m* and its associated variance. In our case, however, the correspondences are initially unavailable and cannot be established on the basis of local image information because the *A* and *B* are too different in appearance. In short, this means that we must rely only on geometrical properties to simultaneously establish the correspondences and estimate the underlying non-linear transform. Since attempting to do this directly for all edge points would be computationally intractable, our algorithm goes through the following two steps:

1. **Coarse alignment:** We begin by only matching graph nodes so that the resulting mapping is a combination of an affine deformation and a smooth non-linear deformation. We initialize the search by randomly picking D correspondences, which roughly fixes relative scale and orientation, and using them to instantiate a Gaussian Process (GP). We then recursively refine it as follows: Given some matches between 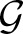
^*A*^ and 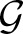
^*B*^ nodes, the GP serves to predict where other 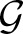
^*A*^ nodes should map and restricts the set of potential correspondences. Among these possibilities, we select the most promising one and use it to refine the GP. Repeating this procedure recursively until enough mutually consistent correspondences have been established and backtracking when necessary lets us quickly explore the set of potential correspondences and recover an approximate geometric mapping.
2. **Fine alignment:** Having been learned only from potentially distant graph nodes, the above-mapping is coarse. To refine it, we also establish correspondences between points that form the edges connecting the nodes in such a way that distances along these edges, which we will refer to as *geodesic* distances, are changed as little as possible between the two graphs. Because there are many more such points than nodes, this would be extremely expensive to do from scratch. Therefore, we constrain the correspondence candidates to edges between already matched nodes and rely on the Hungarian algorithm of Munkres (1957) to perform the optimal assignment quickly.

### 4.2. Results

**Figure 7:**
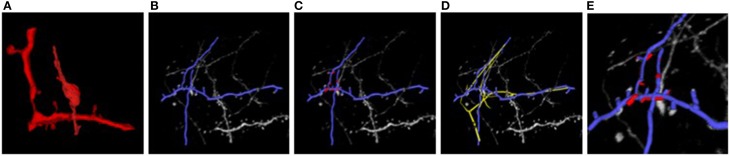
**Light and electron microscopy neuronal trees. (A)** Graph structure extracted from the electron microscopy image stack, in red. **(B)** Segmented light microscope neurons in blue. **(C)** After the non-linear registration process using ATS-RGM, the EM segmented neuron is deformed and aligned over the LM extracted neuron. **(D)** Registration using CPD, in yellow, which falls into a local minimum. **(E)** A zoom over the region where the EM stack has been extracted. The two neurons have been completely aligned. Best viewed in color.

Figure 7 illustrates the two stages of our approach applied to the EM and LM stacks of Figure 1. Even though the two images look extremely different, our algorithm returns a non-rigid deformation that lets us correctly superpose the two stacks. The technique is generic and allows us to correctly align other biological structures, such as blood-vessels networks, that are non-linearly transformed and extracted with different techniques, without having to pre-aligning them and in a manageable amount of time.

## 5. Conclusion

If we are ever to unravel the mysteries of brain function at its most fundamental level, we will need a precise understanding of how neurons connect to each other. With the advent of new high-resolution light and electron microscopes, fast computers, and high-capacity storage media, the data required to perform this task is now becoming available. Electron microscopes (EM) can now provide the nanometer resolution that is needed to image synapses, and therefore connections, while Light Microscopes (LM) see at the micrometer resolution required to model the 3D structure of the dendritic network. Since both the arborescence and the connections are integral parts of the wiring diagram, combining these two modalities is critically important to answer a growing need for automated quantitative assessment of neuron morphology and connectivity.

Here, we have reviewed our approach to addressing this daunting task. Our algorithms are effective at delineating linear structures in LM, segmenting mitochondria and synapses in EM, and putting the results into a unified coordinate systems to produce a joint representation^1^. However, we have so far only modeled small fractions of cells, which only represent minute parts of simple neural circuit. Our challenge therefore is now to scale up our methods so that they can handle much larger volumes, which will involve parallelizing them and using GPUs, instead of CPUs, to massively increase the processing speed.

### Conflict of interest statement

The authors declare that the research was conducted in the absence of any commercial or financial relationships that could be construed as a potential conflict of interest.

## Footnotes

^1^Some of the corresponding software can be downloaded from our lab's website http://cvlab.epfl.ch/software.
